# Controllable Edge Oxidation and Bubbling Exfoliation Enable the Fabrication of High Quality Water Dispersible Graphene

**DOI:** 10.1038/srep34127

**Published:** 2016-09-26

**Authors:** Suyun Tian, Jing Sun, Siwei Yang, Peng He, Gang Wang, Zengfeng Di, Guqiao Ding, Xiaoming Xie, Mianheng Jiang

**Affiliations:** 1 School of Physical Science and Technology, ShanghaiTech University, Shanghai 200031, P.R. China; 2 State Key Laboratory of Functional Materials for Informatics, Shanghai Institute of Microsystem and Information Technology, Chinese Academy of Science, Shanghai 20050, P. R. China; 3 CAS Center for Excellence in Superconducting Electronics (CENSE), Shanghai 200050, China

## Abstract

Despite significant progresses made on mass production of chemically exfoliated graphene, the quality, cost and environmental friendliness remain major challenges for its market penetration. Here, we present a fast and green exfoliation strategy for large scale production of high quality water dispersible few layer graphene through a controllable edge oxidation and localized gas bubbling process. Mild edge oxidation guarantees that the pristine *sp*^2^ lattice is largely intact and the edges are functionalized with hydrophilic groups, giving rise to high conductivity and good water dispersibility at the same time. The aqueous concentration can be as high as 5.0 mg mL^−1^, which is an order of magnitude higher than previously reports. The water soluble graphene can be directly spray-coated on various substrates, and the back-gated field effect transistor give hole and electron mobility of ~496 and ~676 cm^2^ V^−1^ s^−1^, respectively. These results achieved are expected to expedite various applications of graphene.

Oxidation-reduction is the earliest wet process developed for the scalable fabrication of chemically exfoliated graphene, and it remains to be one of the most mature approaches ever developed, due to its versatility, scalability and controllability in terms of surface area, size and thickness^1^,^2^,^3^,^4^. The drawback lies in its compromised quality since lattice defects and functional groups attached to the surface and edges cannot be completely removed during the reduction step^5^,^6^. Strong oxidants, liquid wastes, organic solvents used and dusts also raise environmental concerns^4^.

Weak oxidation or oxidation-free process is very helpful in preserving graphene’s lattice integrity, thus retaining its performance advantages. Extensive efforts were devoted towards this research direction, with many novel processes developed^7^,^8^,^9^,^10^,^11^,^12^. Yang *et al*. demonstrated high yield graphene production by electrochemical exfoliation of graphite with weak oxidation^7^. The molten salt intercalation^8^ and interlayer catalytic exfoliation^9^ produce high quality graphene, at the cost of high processing temperature, and contamination is also unavoidable due to incomplete cleaning by washing. The ultrasonication^10^ and liquid shearing exfoliation^11^ are complete oxidation-free, yielding very high quality graphene, but suffer from low yield of <10% and small lateral size, typically in submicron range.

Water dispersibility is another key performance index for the development of graphene^2^,^13^, releasing largely the environmental constrains of the end user. However, to develop high quality aqueous based graphene, one has to face the dilemma as high quality requires undisturbed lattice while water dispersibility needs defect sites for necessary surface modification. This is because pristine graphene with high lattice quality is hydrophobic in nature^11^,^14^, while the defect sites can be grafted with lots of chemical groups beneficial for stable dispersion in water. Therefore, Li *et al*. developed a method, using remnant structure defects after reduction of graphene oxide, to fabricate water-dispersable graphene^2^. Despite with a low concentration of 0.5 mg mL^−1^, this method remains currently the most common method to obtain aqueous graphene dispersions.

The pursuit of high quality and environmental friendliness leads us to a novel strategy which is to mildly oxidize the graphite only at the edges and to realize exfoliation by bubbles generated by the catalytic reaction at the edges. Weak oxidation only at the edges minimizes lattice damage, while the oxidized edge brings in excellent solubility in water without any surfactants or additives. The water solubility reaches 5 mg mL^−1^, one order of magnitude higher than published results. The aqueous graphene can be directly spray-coated on flexible or rigid substrates, and the high quality was confirmed by carrier mobility measurements on back-gated field effect transistors (FETs). This process developed features good cost-effectiveness and environmental friendliness at the same time. It may be applicable for fabricating other two-dimensional materials with uncompromised quality and water solubility.

## Results

### Edge Oxidation

**Figure 1:**
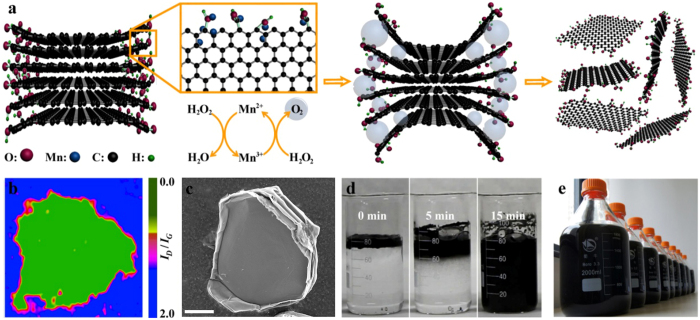
Bubbling exfoliation of edge oxidized graphite for water soluble graphene. (**a**) Schematic diagram of preparation process. Left: oxidation at graphite edges; middle: bubbling and exfoliation; right: dispersion. (**b**) Raman *I*_*D*_/*I*_*G*_ mapping image on a 50 μm × 50 μm graphite, showing most defects located around the edges. **(c)** SEM image of an edge oxidized graphite. (**d**) Digital photographs of bubbling process of 0.5 g precursor in 80 mL bubbling reagent for 0, 5 and 15 min, respectively. (**e**) Digital photographs of 20 L, 2.5 mg mL^−1^ graphene aqueous solution prepared through the bubbling exfoliation. Scale bar, 10 μm (**c**).

Our approach is clearly illustrated in the schematic diagram of Fig. 1a and the bubbling process of Supplementary Video 1. At first, the controllable weak oxidization in the mixture of sulfuric acid and potassium permanganate (KMnO_4_) facilitates the wetting of graphite in water and the edge opening of layered graphite sheets, while keeping most basal planes of graphite intact; Then, the exfoliation is accomplished by interlayer gas bubbling process of the precursor in the mixture of hydrogen peroxide (H_2_O_2_) and ammonia (NH_3_). The weak oxidation on the graphite edges was realized through controlling mass ratios between oxidant KMnO_4_ and natural graphite. The typical mass ratio of KMnO_4_ and natural graphite is 1:1, and the reaction temperature and time is 25 °C and 2 h, respectively. Compared with generally reported Hummers or modified Hummers, as shown in Supplementary Table 1, we employed less oxidant, low temperature and very short reaction time, which results in lower oxygen content and higher C/O ratio of 5.34, as shown in the X-ray photoelectron spectroscopy (XPS) of oxidized graphite data in Supplementary Fig. S1. The oxygen content from XPS is 15.7 at.%, which is double confirmed by CHN elemental analysis. We propose that oxygen mainly locates on the edges and the surface of graphite sheets. The total atomic layer number is more than 2.5 × 10^4^ for a graphite flake with 10 μm thickness, and the surface atomic layers will protect the inner layers from further oxidation while the edges have more defects as “weak” points to be attacked, and the space between graphite layers provides possibility for intercalation from the edges.

The controllable edge oxidation of graphite was verified through Raman spectrum and scanning electron microscopy (SEM) measurements. Figure 1b shows the Raman mapping data on an edge oxidized graphite sheet after removing the surface layers. The intensity ratio of the D band (associated with the edge distortion and structural defects) and the G band (revealing the *sp*^2^ carbon structure)^15^, *I*_D_/*I*_G_, on the edge of the precursor is 0.8–1.5 while its most basal plane shows very low *I*_D_/*I*_G_ (almost 0), which proves that most parts of the sample has intact *sp*^2^ structure, and confirms the efficient and reliable edge oxidation on graphite. The edge oxidation can also be directly confirmed by SEM on the graphite edges before and after before oxidation. As shown in Fig. 1c and Supplementary Fig. S2, the expanded edges of a graphite sheet can be clearly observed due to the enlarged interlayer space by spatial intercalation of oxygen-containing groups. It should be noted that the degree of the edge oxidation can be controlled by adjusting the KMnO_4_ addition. Supplementary Fig. S3 shows the Raman mapping results for oxidized graphite with different mass ratio of KMnO_4_ and natural graphite. With the more KMnO_4_ addition, the weak oxidized area (*I*_D_/*I*_G_ ~ 0) shrunk, and when the mass ratio of KMnO_4_ and natural graphite is 10:1, the oxidation became uniform for the whole flake, which further confirms that the oxidation of a graphite flake stars from the edges and goes into the inner parts gradually.

### Bubbling Exfoliation

After edge oxidation, the bubbling exfoliation immediately and spontaneously took place when the precursor was put into the mixed solution of NH_3_ and H_2_O_2_. A lot of bubbles around precursor came into being, and as a result the graphite sheets floated on water although its density is much higher than that of water, as shown in Fig. 1d and Supplementary Fig. S1. Within 5 minutes, there were lots of thin and dark exfoliated graphene sheets dispersed into water, and the precursor transformed into water-dispersable graphene completely in 15 minutes. After centrifugal separation or just precipitating separation, the stable graphene solution can be obtained. The concentration can be controlled from 0.5 to 5 mg mL^−1^ by just controlling the water amount during the exfoliation or re-dispersion. Especially, the aqueous concentration can be as high as 5.0 mg mL^−1^, which is an order of magnitude higher than previously reports^2^,^13^,^14^. The typical yield of 1–6 atomic layer graphene of this method is 55.2%, by calculated the weight ratio of obtained graphene powder (collected from the stable graphene solution) and the initial natural graphite. It can be concluded that the spontaneous bubbling exfoliation is fast, efficient and environmental friendly. As shown in Supplementary Fig. S4, the volume of the freeze-dried water-dispersable graphene is much larger than that of the precursor of edge oxided graphite, which directly confirms the efficient exfoliation. The NH_3_ and H_2_O_2_ can be removed in the liquid before graphene collection, or by heating the dry products. The freeze-dried water re-dispersed graphene show excellent re-dispersibility in water, N,N-Dimethylformamide (DMF) and N-methyl-2-pyrrolidone (NMP) (Supplementary Fig. S5 and Supplementary Fig. S6), keeping for 15 days, further indicating the advantage of this method. At last, this bubbling exfoliation and dispersion method can be enlarged easily. As shown in Fig. 1e, 20 L aqueous dispersion of water-dispersable graphene with the concentration of 2.5 mg mL^−1^ was easily obtained with the reaction setup in Supplementary Fig. S7.

### Characterization

**Figure 2:**
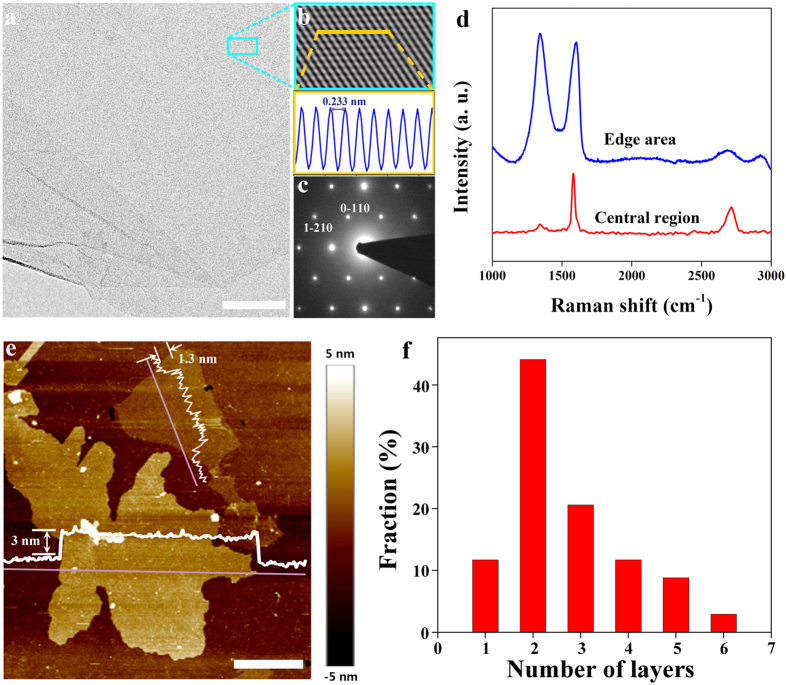
Characterization of graphene. (**a**) TEM and (**b**) local enlarged HR-TEM image of a graphene sheet. (**c**) SAED diffraction pattern of graphene membrane in (**b**). (**d**) Raman spectra on the edge and middle of a graphene sheet. (**e**) AFM image and height profile of graphene deposited on the SiO_2_ substrate. The thickness was ~1.3 and 3 nm. (**f**) Thickness distribution of water soluble graphene. Scale bar, 100 nm (**a**) and 2 μm (**e**).

To evaluate the quality of bubbling-exfoliated graphene, the samples were characterized by SEM, transmission electron microscope (TEM), atomic force microscopy (AFM), X-Ray Diffraction (XRD), Raman, XPS and Fourier transform infrared spectroscopy (FT-IR). The SEM image of Supplementary Fig. S8 shows graphene sheets with a typical area of 3 μm × 2 μm, which is comparable with that of reduced graphene oxides^16^,^17^ and electrochemically exfoliated graphene^18^, and is much larger than ultrasonication^10^ or liquid phase shearing^11^. TEM image of Fig. 2a shows the wrinkles of a graphene thin sheet, suggesting a good flexible and ultrathin nature. High resolution TEM (HR-TEM) image of Fig. 2b shows the distinct crystallinity with lattices of 0.25 nm, which can be attributed to the (1120) lattice^14^,^18^. The single-layer, bilayer, trilayer and few-layer graphene sheets are determined by the dark lines in the folded regions (Supplementary Fig. S9). According to HR-TEM image analysis, the products are predominately less than 6 atomic layers. Selected area electron diffraction pattern (SAED) (Fig. 2c) indicates typical six fold symmetry structure of graphene with same strong diffraction from the (1–210) plane and from the (0–110) plane, indicating the high crystallinity of single and bilayer graphene sheet^19^. Moreover, the Raman in Fig. 2d and XRD in Supplementary Fig. S10 further confirmed the formation of thin graphene. The low *I*_*D*_*/I*_*G*_ ratio (0.13) in Raman data on the central region of a graphene basal plane indicates the highly crystallized *sp*^2^ structure of the products. Compared with the *I*_*D*_*/I*_*G*_ ratio of precursor (0.12), the slight change implies no additional oxygen-containing groups formed in exfoliation progress. Moreover, the symmetrical 2D band (*ca.* 2700 cm^−1^) suggests the sample is the complete few layer graphene rather than the multilayered graphite (with asymmetrical 2D band)^15^,^20^. The typical AFM image is shown in Fig. 2e, indicating two graphene sheets with irregular shape and a lateral size of several microns. The thickness of the graphene sheets 1.3 and 3.0 nm, calculated from the height difference between the graphene and the substrate, corresponding to approximately 3 and 6 atomic layer thickness, respectively^12^,^18^. According to a lot of AFM tests, the thickness distribution of the obtained graphene sheets is shown in Fig. 2f. The thickness mainly distributes in 0.5–3.0 nm, which is consistent with HR-TEM data. In addition, the XPS and FTIR measurements were undertaken to characterize the functional groups on typical graphene. XPS survey spectrum shows a predominant narrow graphitic C 1s peak at *ca.* 284.2 eV (83.75 at. %), along with an O 1s peak at *ca.* 532 eV (16.25 at. %), as shown in Supplementary Fig. S11a. Well-fitted C 1s XPS spectrum is shown in Supplementary Fig. S11b. The C 1s XPS spectrum can be divided into three different peaks, which correspond to the signals of *sp*^2^-hybridised carbon (C=C, 284.86 eV, 67.4%), epoxy/hydroxyls (C–O, 287.0 eV, 24.2%) and carboxyl/carbonyl groups (C=O, 288.0 eV, 8.4%)^21^. The O 1s XPS spectrum can be divided into two different peaks (Supplementary Fig. S11c), and oxygen-containing groups are doubly confirmed by FT-IR results (Supplementary Fig. S12).

According to the synthesis procedure and measuring results, the bubbling exfoliated graphene should have good quality without the needs of reduction since most part of the basal plane is close to pristine graphene, differentiated from the uniform oxidation of graphite through the typical Hummers method. We further demonstrate the advantages of our preparing strategy through good water solubility, conductive film on polymer, and FET performance. Supplementary Fig. S13 presents the Tyndall effect when a laser beam is passing through a homogeneous graphene aqueous dispersion, suggesting the uniform graphene dispersion in water. Supplementary Fig. S14a shows a typical ultraviolet-visible (UV-Vis) spectrum of this dispersion, where the peak located at 230 nm can be attributed to the π-π* electron transmission of C=C bonds, and the shoulder peak at 300 nm can be attributed to the n–π* electron transmission of C=O bonds^22^,^23^,^24^. The value (A) of the peak at 300 nm was selected to show the stability of graphene in aqueous phase. As shown in Supplementary Fig. S14b, no obvious change can be observed after 8 days, indicating good stability of our products. This can be due to the edge oxygen-containing groups of the water-dispersable graphene. Supplementary Fig. S15 shows the stability of water-dispersable graphene aqueous solution with different oxidation degree. Obviously, the water-dispersable graphene with high oxidation degree shows better stability in water. This illustrates the main role of oxygen-containing groups of water-dispersable graphene for stabilized dispersion in water.

The water-dispersable graphene can be directly spray coated on several substrates. 1.0 mL of water-dispersable graphene ink can be spray coated on a 50 cm^2^ (10 cm × 5 cm) polyethylene terephthalate (PET) substrate through a commercial liquid sprayer (Supplementary Fig. S16). After the initial drying of the film at 80 °C, the coating is further dried at 150 °C for 0.5 h. The sheet resistance of obtained film is 2196 ohms ◻^−1^ with a transmittance of 65.9%. SEM image (Supplementary Fig. S17) shows the graphene ultrathin sheets excellently spreading on the PET substrate. The sheet resistance is much lower than most reported rGO (mostly larger than 10000 ohms ◻^−1^) and close to CVD graphene (500–1500 ohms ◻^−1^), as summarized in Supplementary Fig. S18. Good ohmic contact between well-crystallized graphene sheets results in the good electrical conductivity of the graphene coating. The coating thickness or transparence can be adjusted by spraying procedure or the graphene concentration in water. Supplementary Fig. S19 shows the relationship between conductivity and transparence of obtained graphene film on PETs. The sheet resistance of the water soluble graphene film increased with the increased transmittance (450 nm). The sheet resistance is 2169, 762.7, 418.0, 380.5 and 119.6 ohms ◻^−1^ when the transmittance is 65.9%, 42.9%, 32.5%, 29.3% and 5.56%, respectively, which means that the graphene can be easily spray coated onto polymer substrate, and can be used to construct the flexible conductive film. The inset in Supplementary Fig. 19 shows that the graphene coating layer on a paper can be directly employed as flexible conductor.

**Figure 3:**
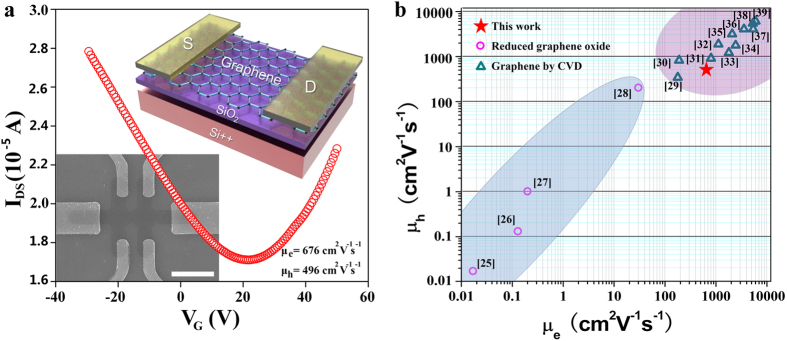
Applications of water soluble graphene thus formed. (**a**) I_DS_-V_G_ curve of water soluble graphene transistors at V_D_ = 150 mV, the insert shows the schematic diagram and SEM image of the FET device. **(b**) The electron and hole mobility statistics of graphene based FET in previous reports (black circles: based on reduced graphene oxide, red circles: based on CVD graphene) and this work (red star). Scale bar, 10 μm (**a**).

To determine the transport properties of the synthesized graphene films, back-gated graphene field effect transistors FETs were fabricated on 300 nm SiO_2_/Si substrates. The inset of Fig. 3a shows the schematic diagram and SEM image of the fabricated FETs. Highly reproducible transfer characteristics (I_DS_-V_G_) of the FETs measured at room temperature under ambient conditions. The typical I_DS_-V_G_ curve measured at a V_DS_ of 150 mV shows that the gate can cause either hole or electron conduction. The Dirac point of the FETs shifts slightly to a positive gate at V_G_ ~ 25 V, demonstrating light p-type hole doping performance. According to the two slopes of the linear regions on both sides of the V-shaped curve, the hole mobility is μ_h_ ~ 496 cm^2^ V^−1^ s^−1^ and the electron mobility is μ_e_ ~ 676 cm^2^ V^−1^ s^−1^. As shown in Fig. 3b, the electron mobility of our water-dispersible graphene is much higher than that of graphene obtained by oxidation-reduction approaches^25^,^26^,^27^,^28^, and is also comparable to that of graphene films fabricated through high temperature CVD approaches^29^,^30^,^31^,^32^,^33^,^34^,^35^,^36^,^37^,^38^,^39^. Considering that the spray coating progress is violent and inevitably introduces defects, impurities, wrinkles or overlaps, the performance of the FETs may be underestimated.

### Mechanism

It is necessary to discuss the exfoliation mechanism to further understand this efficient approach. According to Fig. 1b,c and Supplementary Figs S2 and S3, the Raman and SEM results clear show the edge intercalation and oxidation. Around the edges, the Mn ion residues were detected by XPS measurement (Supplementary Fig. S20) and EDS mapping during TEM test (Supplementary Fig. S21). Although the amount of Mn ion residues is very limited, and it is very difficult to be detected by XPS and EDS, these residues play a critical role for the bubbling exfoliation. It is well know that, due to the diversified oxidation states of Mn, the Mn ions are ideal catalysts for the decomposition course of H_2_O_2_^40^, through a catalytic progress. For demonstration, we use Mn^3+^ as an example as shown in Fig. 1a:
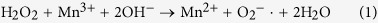

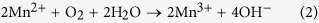

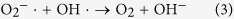


In fact, all kinds of Mn salts can catalytically accelerate the bubbling. To verify this, we fabricate graphite oxide through the Staudenmaire method using KClO_4_ as oxidant to avoid the Mn impurities. Then, we added graphite oxide, graphite oxide + MnSO_4_ (0.5 g + 10 mg), graphite oxide + MnO_2_ (0.5 g + 10 mg), graphite oxide + KMnO_4_ (0.5 g + 10 mg) into the 80 mL H_2_O_2_/NH_3_ solutions, respectively, as shown in Supplementary Fig. S22. Obviously, there were no bubbles when graphite oxide itself was added into the solution, and the mixture became green. But when both graphite oxide and MnSO_4_ were added, as well as graphite oxide + MnO_2_ and graphite oxide + KMnO_4_, large amount of bubbles generated immediately, and the color of the mixture gradually changed to black. These results confirm that the Mn is a key factor for the bubbling exfoliation, no matter what its valence state.

In addition to the Mn ions, the catalytic decomposition progress of H_2_O_2_ requires the alkaline medium^41^,^42^,^43^. Thus, the NH_3_ also plays an important role in bubbling progress. NH_3_, not NaOH or KOH, was chosen since it can be easily removed in the obtained products. The exfoliation progressed in different mediums confirmed the importance of NH_3_ intuitively. As shown in Supplementary Video 2, the precursor is put into different mediums. The volume of H_2_O_2_ is 5, 4, 3, 2, 1, 1, 1, 1 and 0 units, respectively from left to right. At the same time, the volume of NH_3_ is 0, 1, 1, 1, 1, 2, 3, 4 and 5 units, respectively from left to right (1 unit volume is 10 mL). The drastic bubbling process can be observed only with the present of both H_2_O_2_ and NH_3_. However, no bubble can be observed without the present of NH_3_, which demonstrated its assisted catalysis effect. Thus, Mn ions, H_2_O_2_ and NH_3_ are the key factors for the bubbling exfoliation. The H_2_O_2_ is the most important factor, with the assistance of Mn ions and NH_3_. The O_2_ bubbles continually generate in the interlamination of graphite edges, which facilitates continuous and fast exfoliation of the precursor into water-dispersable graphene (Supplementary Fig. S23). Supplementary Fig. S24 shows the TEM images of typical edge oxidized graphite/graphene sheets under different reaction time (5, 10, 15 min). Thick graphene sheet, ~20 atomic layers, was detected in the bubbling solution at the initial reaction stage (<5 min), and 4–5 atomic layer graphene came into being after 10 min bubbling exfoliation, and at last, the main product is thinner graphene with just 2–3 atomic layers in thickness.

In order to further demonstrate the bubbling exfoliation mechanism intuitively, highly oriented pyrolytic graphite (HOPG) was selected as the raw material. HOPG was oxidized under the same reaction conditions as natural graphite to obtained edge oxidized HOPG. Before the bubbling exfoliation, the HOPG appearance had no obvious change after the weak oxidation. When it was put into the mixture of H_2_O_2_ and NH_3_ (the volume of H_2_O_2_ and NH_3_ is 40 mL and 10 mL, respectively), the bubbles generated immediately at the edges and HOPG expanded lengthways, as shown in Supplementary Video 3. At last, the longitudinal dimension is 20 times than that before exfoliation (Supplementary Fig. S25). These phenomena on HOPG directly confirm the weak oxidation and bubbling exfoliation from the edges of precursors. These results indicate that it is the Mn ion residues in the precursor catalytically decompose H_2_O_2_ with the help of NH_3_ to *in-situ* continuously generate bubbles, and that the bubbling exfoliation takes place between all the graphite layers from the edges, not a layer-by-layer model. It is the exfoliation model that helps the fast spontaneous exfoliation of edge-oxidized precursor into high quality graphene in 15 minutes.

## Discussion

For graphite to graphene, we employed a new, simple, mild, mild, fast and scalable spontaneous exfoliation approach. It is based on the weak edge oxidation with the controllable dosage of KMnO_4_ and the *in-situ* continuous bubbling exfoliation with the catalytic H_2_O_2_ decomposition. The unique graphene, with edge oxidized and most basal plane intact, has excellent water solubility with extremely high concentration of 5 mg mL^−1^, good conductivity without the need of reduction, large lateral size of several microns and 1–6 atomic layer in thickness. This approach not only provides a new strategy for graphene synthesis, but also paves the way for large scale application of graphene in aqueous mediums, such as water-based green or functional coatings.

## Methods

### Materials

Natural graphite powder (45 μm) was purchased from Huatai Lubricant sealing S&T Co.Ltd (Qingdao China). H_2_SO_4_ (98%), H_2_O_2_ (30.0 wt. %) and NH_3_ (25 wt. %) were purchased from Lingfeng (Shanghai China). KMnO_4_ (99%) was purchased from Jianglaibio (Shanghai China). Highly oriented pyrolytic graphite (HOPG) was purchased from Shanghai NTI Co. Ltd (Shanghai China). The water used throughout all experiments was purified through a Millipore system.

### Preparation of water-dispersable grapheme

For the preparation of weak oxidized graphite, 10 g graphite was mixed and agitated with 200 mL concentrated H_2_SO_4_ for 30 minutes at 25 °C in 500 mL double-layer beaker with a cooling system based on Julabo F20. 10 g potassium permanganate (KMnO_4_) was added slowly to the mixture in 30 minutes. After the whole KMnO_4_ was added, the reaction was kept for 2 hours. Next, 100 mL deionized water was added and stirred continually for 2 hours. Finally, 10 mL (H_2_O_2_) was added to the mixture and reacted for 30 minutes. The final liquid was washed with the deionized water by a vacuum filtration and frozen drying in order to obtain the edge-oxidized graphite. The precursor was put into the mixing liquid of NH_3_:H_2_O_2_:H_2_O (1:1:5) for 30 minutes to finish the bubbling exfoliation. The exfoliated graphene was centrifuged, filtered and frozen dried. The yield was obtained by calculating the mass ratio of exfoliation graphene after frozen dried and the raw natural graphite.

### Controllable edge oxidation

To control the edge oxidation, we used the same chemicals (H_2_SO_4_, KMnO_4_) with same dosages except various graphite additions (1, 5, 10 g). The mass ratio of KMnO_4_ and graphite is 10:1, 2:1 and 1:1, respectively.

### Experiments on HOPG

HOPG (0.3 g) is mixed and agitated with concentrated H_2_SO_4_ (6.0 mL, for 30 minutes). Next, potassium permanganate (0.3 g) is added slowly to the mixture during 30 minutes (note: some ice is placed out of the beaker for ensuring the reaction in room temperature). After the whole KMnO_4_ is added, the reaction is kept for 2 hours and then deionized water (3.0 L) is added and stirred continually for 2 hours. Finally, H_2_O_2_ (0.6 mL) also is add to the mixture reacting for 30 minutes. The final liquid is washed with the deionized water by a vacuum filtration and frozen drying in order to obtain the dry product. The edge oxidized HOPG (0.3 g) is put into the mixing liquid of NH_3_:H_2_O_2_:H_2_O (1:1:5) and frozen drying. The high quality soluble graphene sheet would be obtained.

### Large scale preparation

For the preparation of weak graphite oxide, the graphite (G) (500 g) is mixed and agitated with concentrated H_2_SO_4_ (10 L, for 30 min). Next, potassium permanganate (500 g) is added slowly to the mixture during 30 minutes (note: some ice is placed out of the beaker for ensuring the reaction in room temperature). After the whole KMnO_4_ is added, the reaction is kept for 2 hours and then deionized water (5 L) is added and stirred continually for 2 hours. Finally, H_2_O_2_ (500 mL) also is add to the mixture reacting for 30 minutes. The final liquid is wash with the deionized water by a vacuum filtration and frozen drying in order to obtain the dry product. The 500 g edge oxidized graphite is put into the mixed solution of NH_3_:H_2_O_2_:H_2_O (1:1:5) to exfoliate the water soluble graphene. After 0.5 h, the homogeneous dispersion liquid of water soluble graphene is obtained. The yield is 52.2% (calculated from graphite).

### Characterization

Atomic Force Microscopy (AFM) data were obtained in a Bruker Dimension Icon with a Nanoscope 8.15 in tapping mode. Scanning electron microscopy (SEM S4700, Hitachi Inc.) was used to image the morphology of the samples. Transmission electron microscopy (TEM) measurements were obtained on a Hitachi H-8100 EM (Hitachi, Tokyo, Japan) with an accelerating voltage of 200 kV. X-ray photoelectron spectroscopy (XPS) measurements were carried out using a Thermo ESCALAB 250Xi spectrometer. Raman Spectroscopy with the excitation laser line of 532 nm was performed using a Thermo Fisher DXR Raman Microscope. X-ray diffraction (XRD) patterns were obtained from a X-Ray Diffractometer (Bruker D8 ADVANCE) with a monochromatized source of Cu Kα1 radiation (λ = 0.15405 nm) at 1.6 kW (40 kV, 40 mA). The sheet resistances were measured by a Hall measurement system (Accent HL5500). Ultraviolet–visible spectra were obtained using a Varian Cary 300 Bio UV-visible spectrophotometer. Fourier transform infrared spectra were recorded on a PE Paragon 1000 spectromer (film or KBr disc).

### Electrical property evaluation

The graphene films were spray coated to highly doped p-Si substrate with a 300 nm thick thermal oxide, followed by deposition of source and drain electrodes with Au/Ti (50/10 nm) by electron beam evaporation. Afterwards, another standard photolithographic step employing inductively coupled plasma (ICP) was used to pattern the graphene to form a field-effect transistor with a channel length of 8 μm and width of 2 μm. To impove the contact of the back-gated GFETs device, annealing was performed in Ar (500 sccm) and H_2_ (10 sccm) at 300 °C for 8 h in a tube furnace. The back-gated GFETs were characterized under ambient conditions using the Agilent (B1500A) semiconductor parameter analyzer. The mobility was extracted using the following equation (S1):
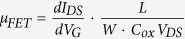
where *L* and *W* are the channel length and width, respectively, *C*_*ox*_ is the gate oxide capacitance (11 nF cm^−2^), V_DS_ is the source drain voltage, I_DS_ is the source drain current, and V_G_ is the gate voltage. The linear regime of the transfer characteristics was used to obtain *dI*_*DS*_*/dV*_*G*_.

## Additional Information

**How to cite this article**: Tian, S. *et al*. Controllable Edge Oxidation and Bubbling Exfoliation Enable the Fabrication of High Quality Water Dispersible Graphene. *Sci. Rep.* **6**, 34127; doi: 10.1038/srep34127 (2016).

## Supplementary Material

Supplementary Information

Supplementary Video 1

Supplementary Video 2

Supplementary Video 3
