# Supplementary material for: Controllable Edge Oxidation and Bubbling Exfoliation Enable the Fabrication of High Quality Water Dispersible Graphene
Source: Sci Rep. 2016 Sep 26;6:34127. doi: 10.1038/srep34127 (PMC5036305; doi:10.1038/srep34127)
Supplement: Supplementary Information [file srep34127-s1.docx]

**Supporting information**

**Controllable Edge Oxidation and Bubbling Exfoliation Enable the Fabrication of High Quality Water Dispersible Graphene**

Suyun Tian^1,2, #^, Jing Sun^2,#^, Siwei Yang^2^, Peng He^2^, Gang Wang^2^, Zengfeng Di^2^, Guqiao Ding^2,3,*^, Xiaoming Xie^1,2,*^, Mianheng Jiang^1, 2^

^1^ School of Physical Science and Technology, ShanghaiTech University, Shanghai 200031, P.R. China

^2^ State Key Laboratory of Functional Materials for Informatics, Shanghai Institute of Microsystem and Information Technology, Chinese Academy of Science, Shanghai 20050, P. R. China

^3^ Shanghai SIMBATT Energy Co., LTD, Shanghai, 201821, P. R. China

*Corresponding authors: Prof. Guqiao Ding, gqding@mail.sim.ac.cn

Prof. Xiaoming Xie, xmxie@mail.sim.ac.cn

^#^ These authors contributed equally to this work

**Supplementary Table 1: A comparison of our approach with Hummers and modified Hummer methods (M-1, M-2 and M-3)**

|  | Hummers  [1] | M-1 (1999)[S15] | M-2 (2004)[S16] | M-3 (2010)[S17] | This work |
| --- | --- | --- | --- | --- | --- |
| KMnO_4_ : graphite | 3:1 | 3:1 | 4.5:1 | 6:1 | 1:1 |
| Temparature (^o^C) | 98 | 80 ^a^; 35 ^b^ | 20 | 50 | 25 |
| Reaction time (h) | 2-10 | 6 ^a^; 2 ^b^ | 120 | 12 | 2 |
| C/O ratio | 2.25 | 2.2 | 1.8 | - | 5.34 |

^a^ Graphite powder (20 g), H_2_SO_4_ (30 mL), K_2_S_2_O_8_ (10 g), and P_2_O_5_ (10 g）at 80 ^o^C for 6 h. ^b^ Oxidized graphite powder (20 g), H_2_SO_4_, KMnO_4_, at 35 ^o^C for 2 h.


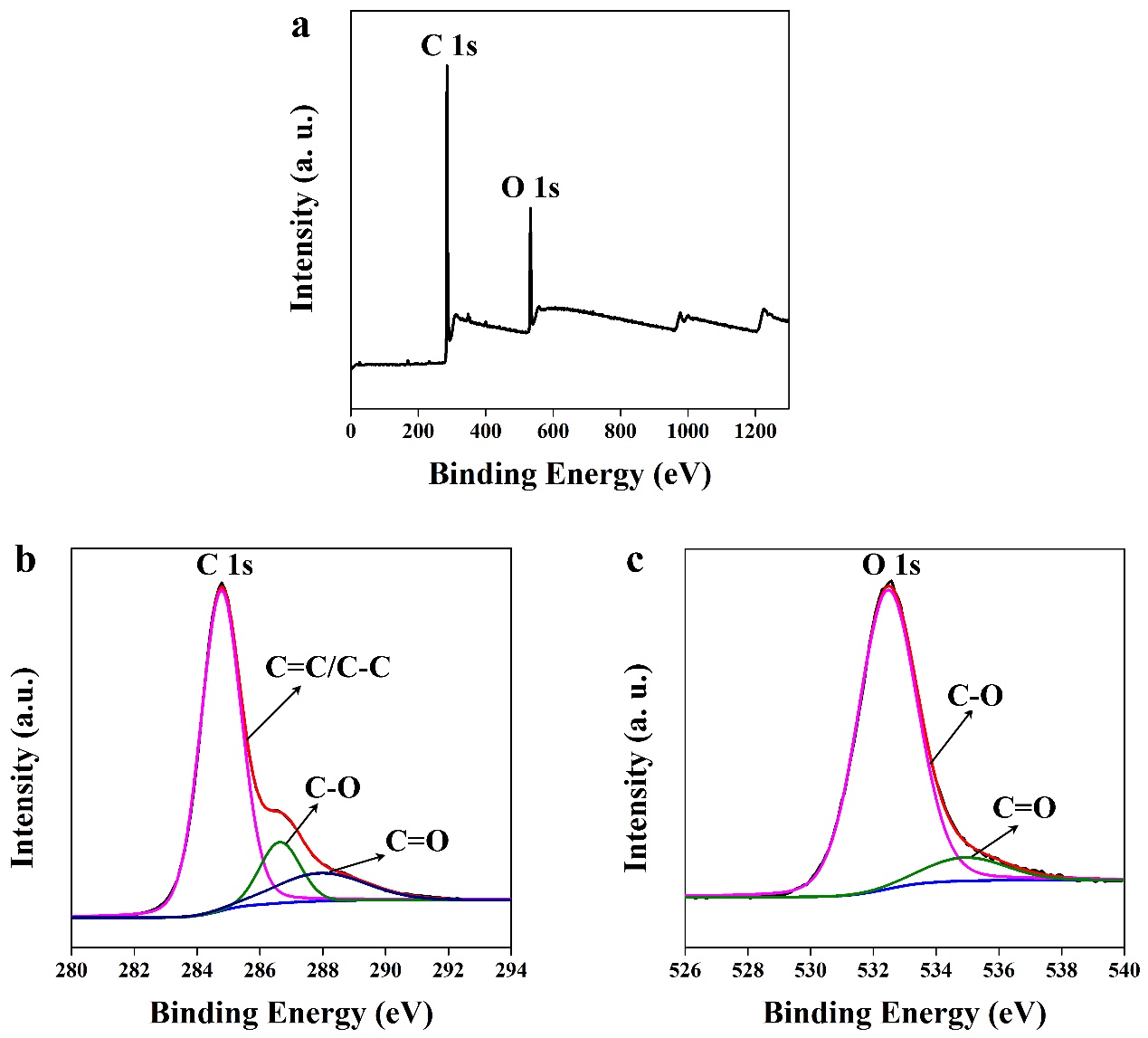


**Figure S1: XPS results of precursor (edge oxidized graphite).** (a) XPS survey spectrum of precursor which shows a C 1s peak at ca. 284.2 eV along with an O 1s peak at ca. 532 eV. (b) C 1s XPS spectra of precursor which can be divided into three different peaks (C-C/C=C, 284.86 eV; C–O, 287.0 eV and C=O, 288.0 eV). (c) O 1s XPS spectra of precursor singlet at 532.5 eV which can be due to the C=O bonding state.


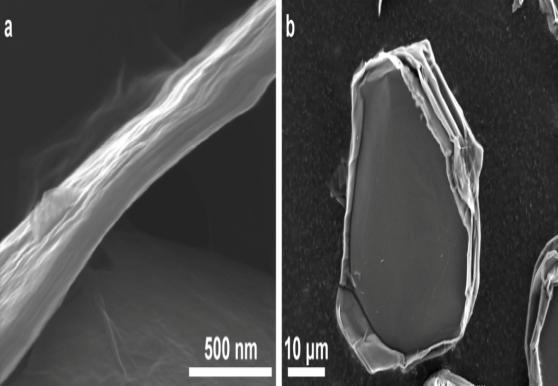

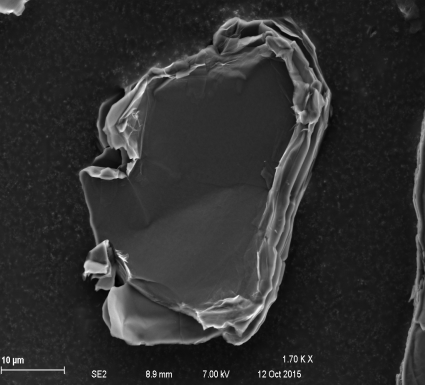


**10 μm**

**b**

**Figure S2: SEM images of** **natural graphite without oxidation.** (a) Cross-sectional view. (b) Top view.


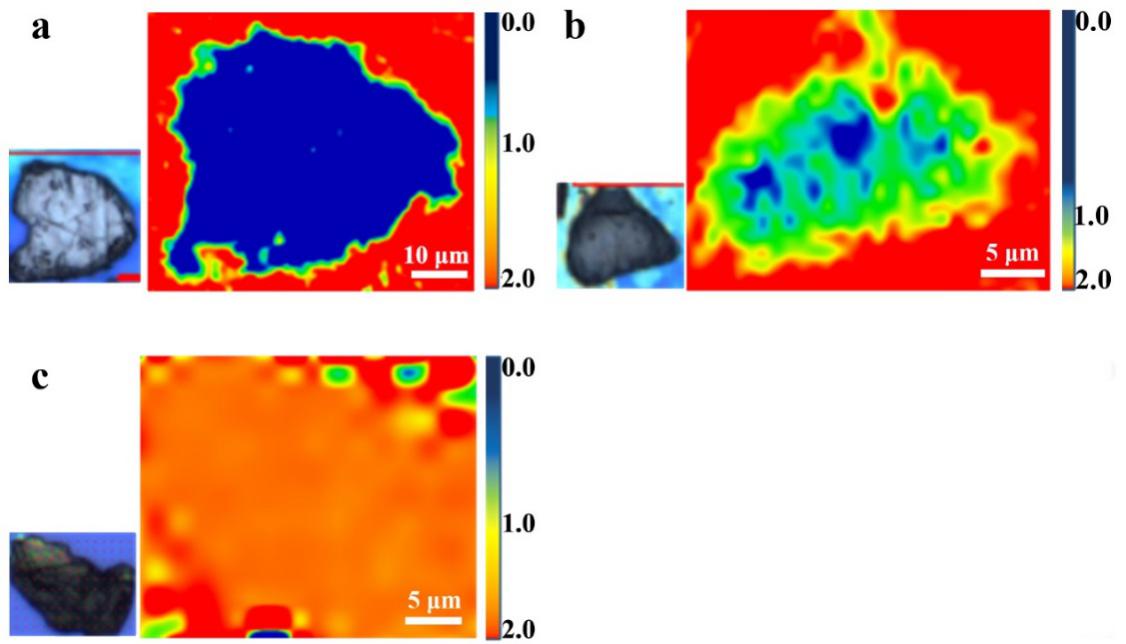


**Figure S3: Edge oxidation control.** Raman map image and corresponding digital photograph of oxidized graphite obtained with mass ratio of KMnO_4_: graphite (a) 1:1, (b) 2:1, and (c) 10:1.


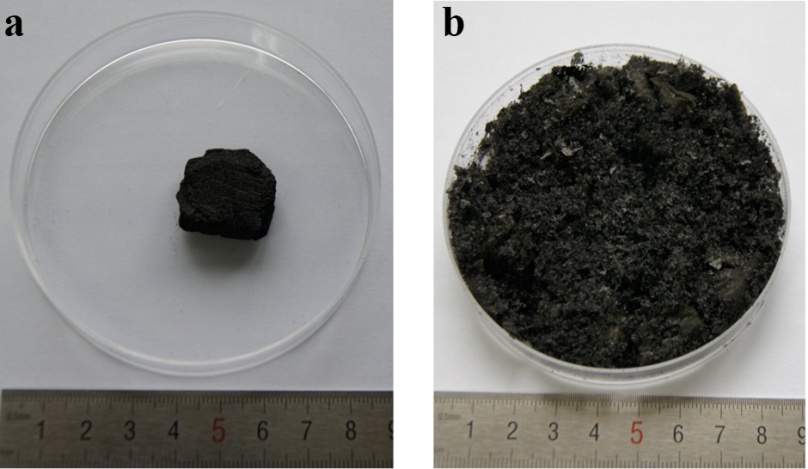


**Figure S4: Water soluble graphene.** (a) Digital photograph of 0.5 g precursor and (b) freeze-dried water soluble graphene obtained from 0.5 g precursor. Through gentle and short time exfoliation, the volume of the freeze-dried water soluble graphene increases explosively compared to that of the precursor.


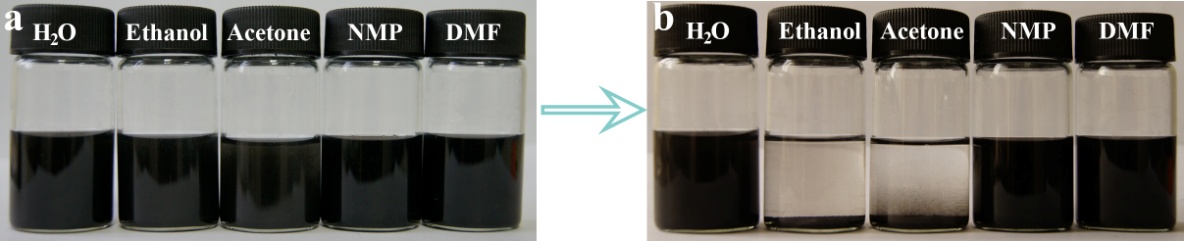


**Figure S5: Re-dispersibility of water soluble graphene in different solvents.** (a) Digital photograph of freeze-dried water soluble graphene re-dispersed in water, ethanol, acetone, NMP and DMF with the concentration of 1.0 mg mL^-1^. (b) Digital photograph of freeze-dried water soluble graphene re-dispersed in water, ethanol, acetone, NMP and DMF after staying for 2 day. The water soluble graphene show excellent re-dispersibility in straight polar solvents (water, DMF and NMP) but show poor re-dispersibility in other solvents (ethanol and acetone).This can be due to the strong intermolecular interaction between the of oxygen-containing groups water soluble graphene and straight polar solvents.


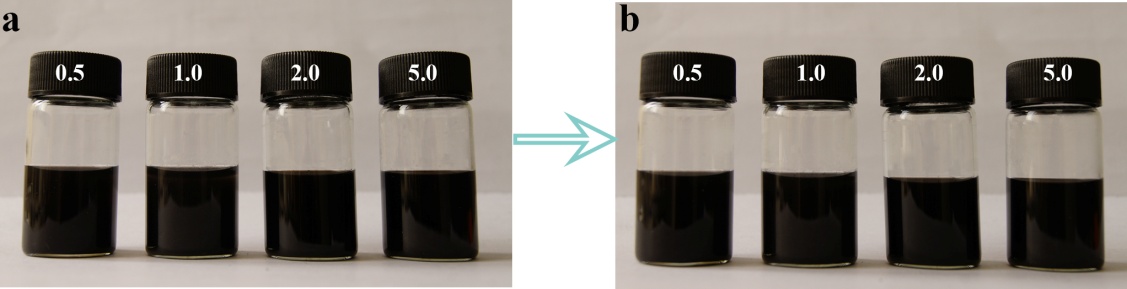


**Figure S6: Re-dispersibility of water soluble graphene in water.** (a) Digital photograph of freeze-dried water soluble graphene re-dispersed in water with concentrations of 0.5, 1.0, 2.0 and 5.0 mg mL^-1^, respectively. (b) Digital photograph of solutions after staying for 2 days. No obvious settlement action and aggregation can be observed, which indicates the excellent re-dispersibility of the water soluble graphene.


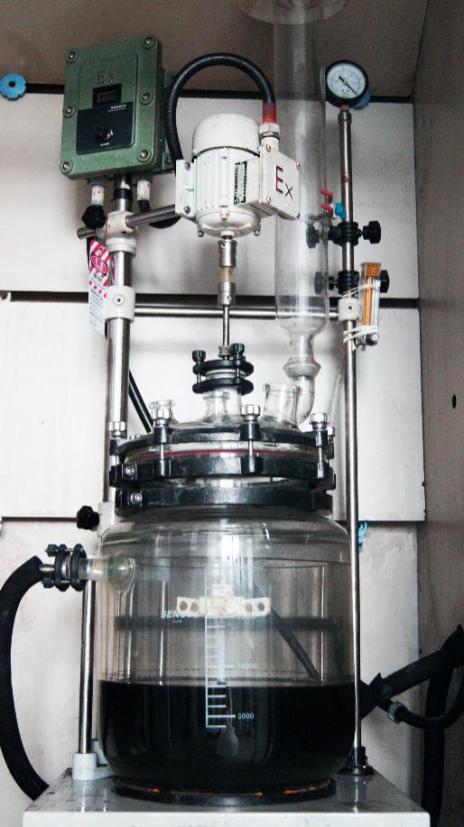


**Figure S7: Digital photograph of large scale preparation equipment of precursor in 20 L reaction setup.**





**Figure S8: SEM image of isolated few-layer graphene with a large area (3 µm×2 µm) on SiO_2_ substance.**


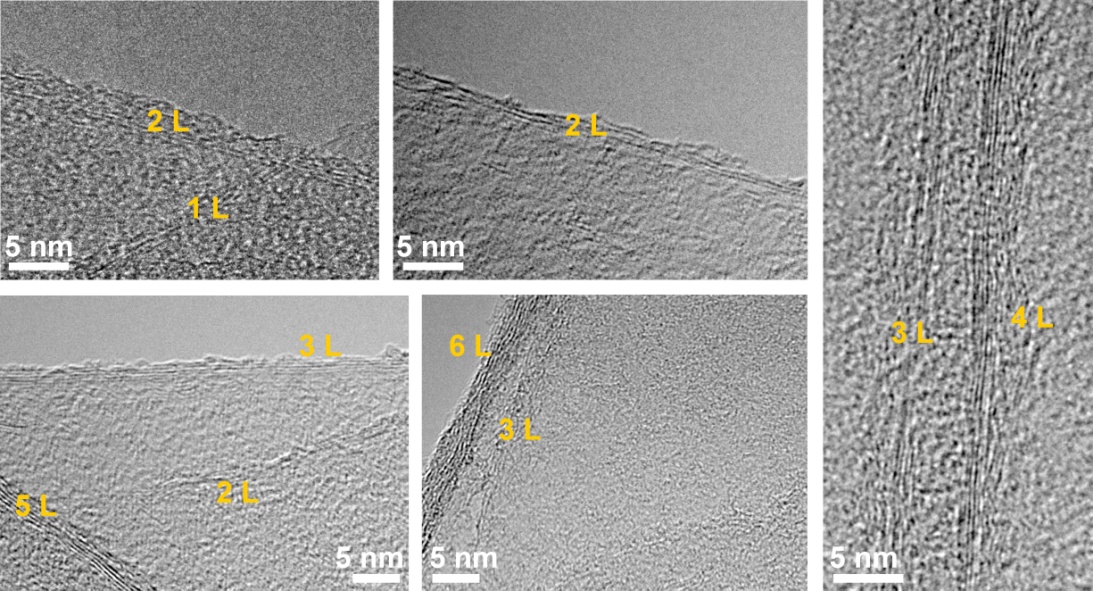


**Figure S9: HRTEM images of the folded edges, indicating single-layer, bilayer, trilayer and few-layer graphene.**


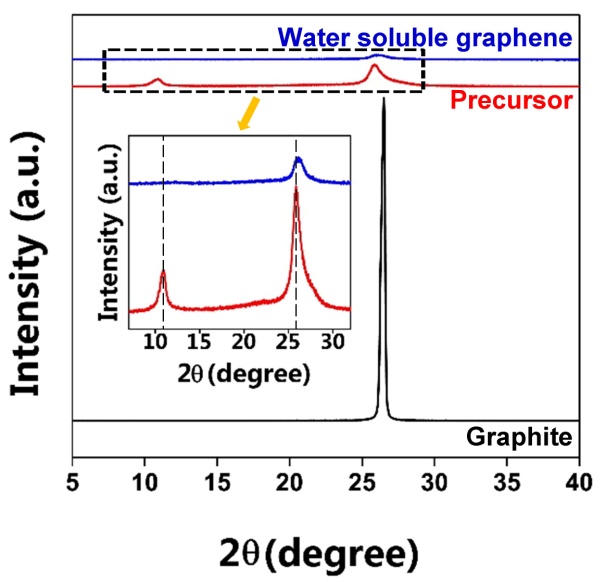


**Figure S10:** **XRD patterns of graphite, precursor and water soluble graphene.** The peak at 10.8° for precursor differing form the others is attributed to a wider inter-layer distance (8.13 Å) between graphene sheet by edge intercalation, and the left shift of peak at 25.8° (002) is due to the complete structure in basal plane of graphene sheet. The peak (002) at 26° for water soluble graphene is corresponding to inter-layer distance (3.415 Å).


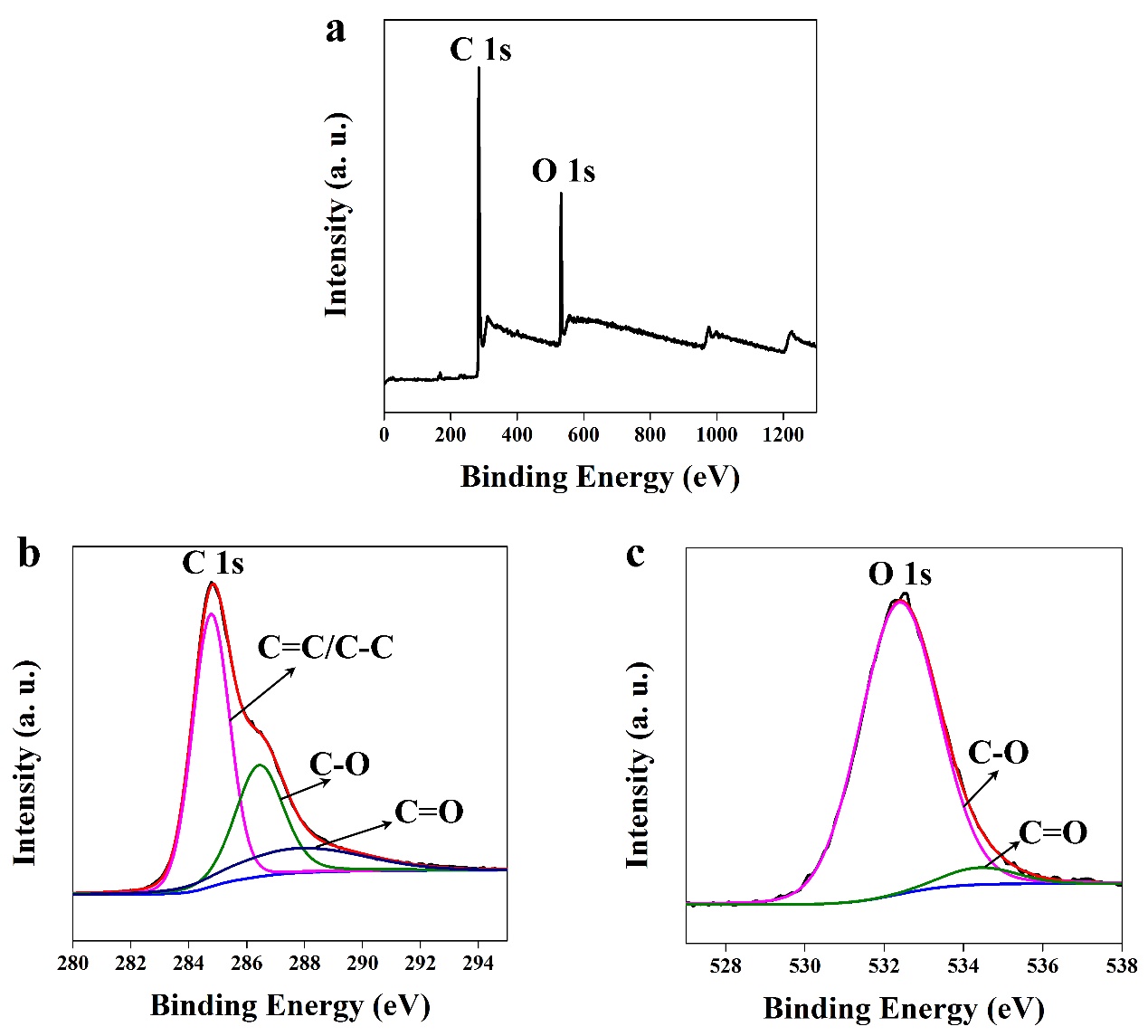


**Figure S11: XPS results of water soluble graphene.** (a) XPS survey spectrum for water soluble graphene which shows a C 1s peak at ca. 284.2 eV, a N 1s peak at ca. 399 eV along with an O 1s peak at ca. 532 eV. (b) C 1s XPS spectra showing three chemical bonding states. (c) O 1s XPS spectra of water soluble graphene showing two chemical bonding states. The peak located at 532.5 and 533.0 eV can be due to the C=O and C-O bonding state, respectively.


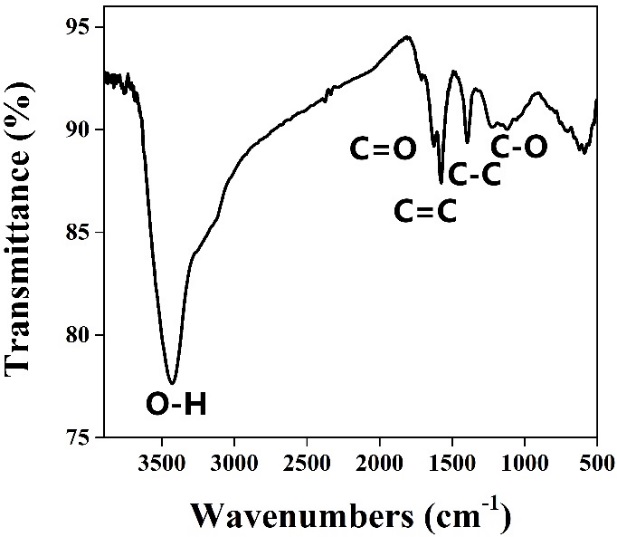


**Figure S12: FT-IR spectrum of water soluble graphene.** The strong peak at 1650 cm^-1^ are assigned to C=C stretching vibrations in aromatic ring structure. The peak at 1450 cm^-1^, characteristic of C-C out-of-plane bending vibrations of benzene nuclei in the aromatic ring skeleton, respectively. The peak at 1700 cm^-1^ correspond to the out-of-plane deformation of C=O stretching vibrations. The peak at 1250 cm^-1^ associates with the C-O vibrations in the benzenoid. Moreover, the peak at 3450 cm^-1^ corresponds to the O-H stretching mode which indicates the presence of secondary hydroxies.


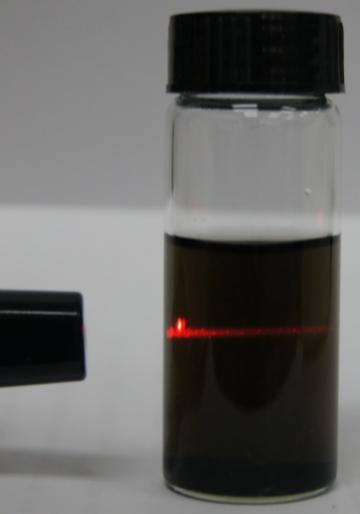


**Figure S13: Digital photograph of 0.5 mg·mL^-1^ homogeneous graphene** **aqueous dispersion which stockpiled for 6 days.** The graphene aqueous dispersion exhibits the Tyndall effect when a laser beam is passing through, suggesting the uniform graphene dispersion in water.


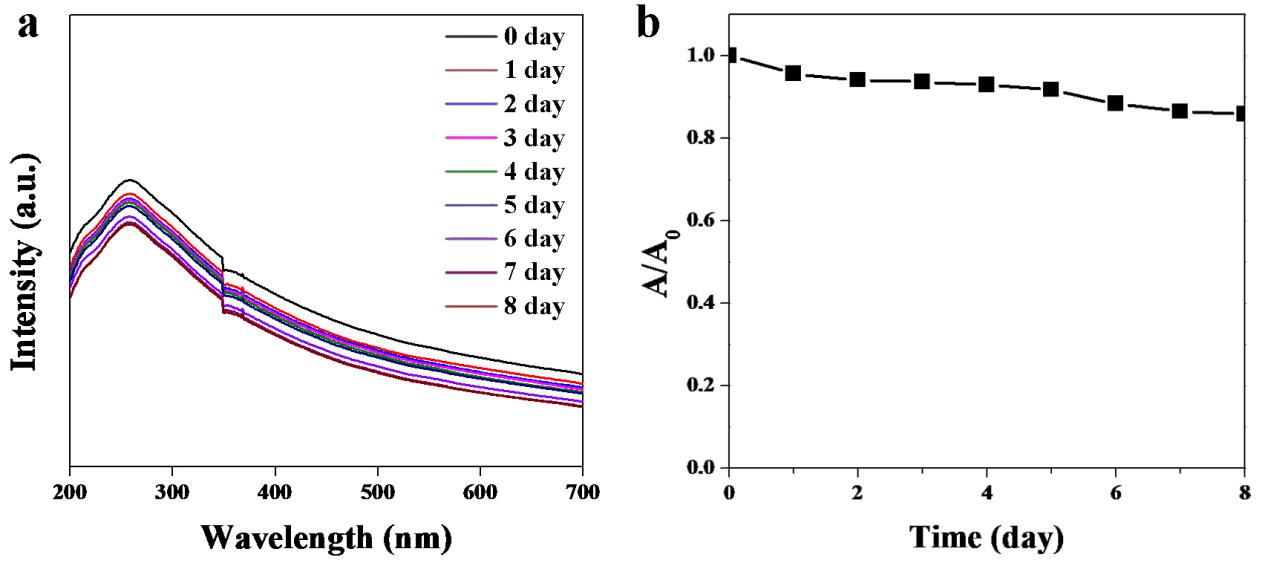


**Figure S14: Stability of water soluble graphene**. (**a**) Stability of typical water soluble graphene aqueous solution. The mass ratio of KMnO_4_ and graphite in preparation process is 1:1. The UV-vis spectra of re-dissolved graphene aqueous dispersion shows slight reduce for 1 - 8 days, indicating the slight settlement action of water soluble graphene thus formed for long time storage. (**b**) Comparison of stability of water soluble graphene (0.5 mg mL^-1^) and reduced graphene (black curve) oxide aqueous (red curve) dispersions.


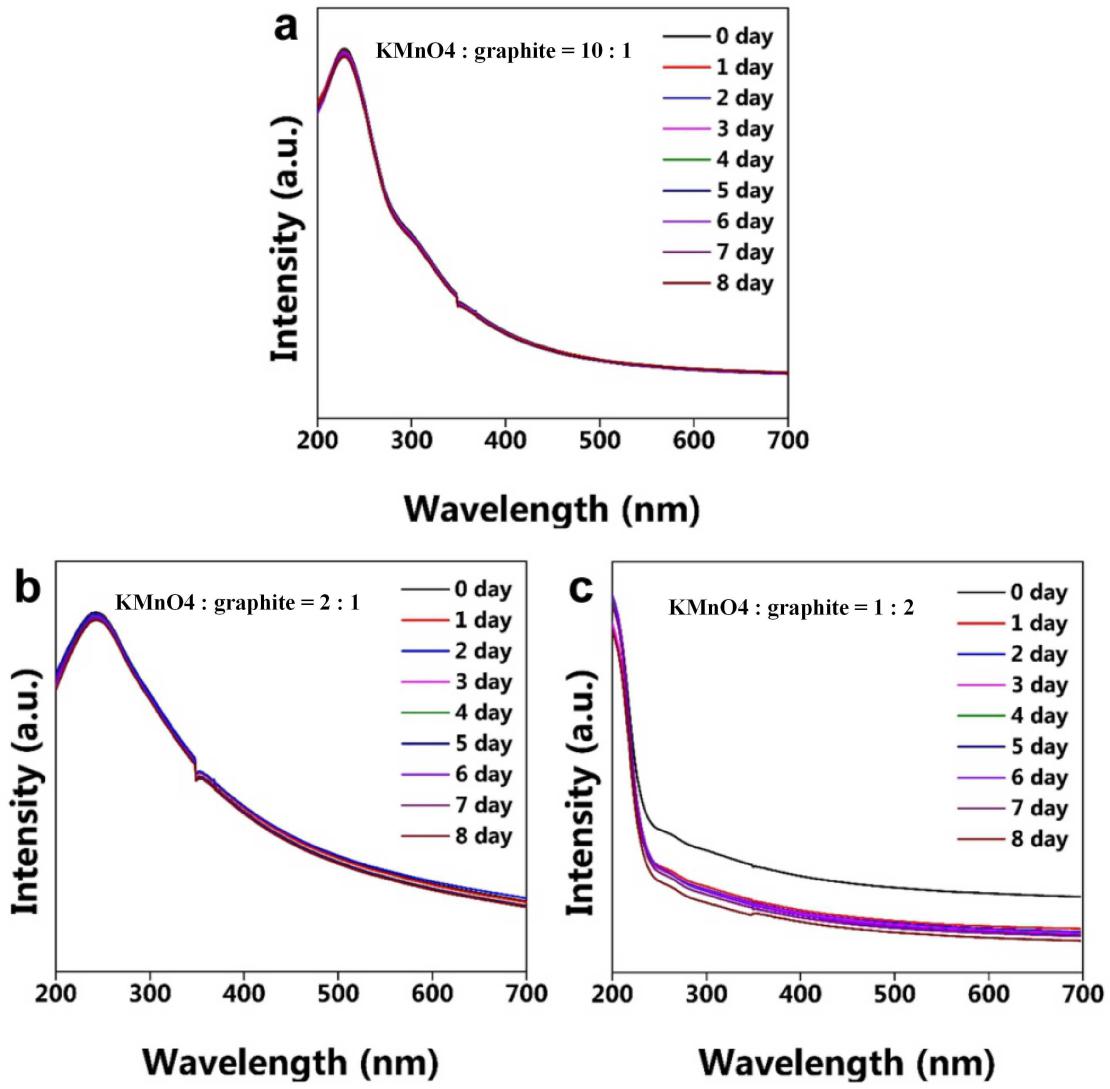


**Figure S15: Stability of graphene aqueous solution with different oxidation degree.** a-c shows the UV-vis spectra of re-dissolved aqueous graphene solutions for 8 days. The mass ratio of KMnO_4_ and graphite in preparation process is (a) 10:1, (b) 2:1 and (c) 1:2. Obviously, the water soluble graphene with high oxidation degree shows better stability in water. This illustrates that the main role of oxygen-containing groups on graphene is to help its dispersion in water.


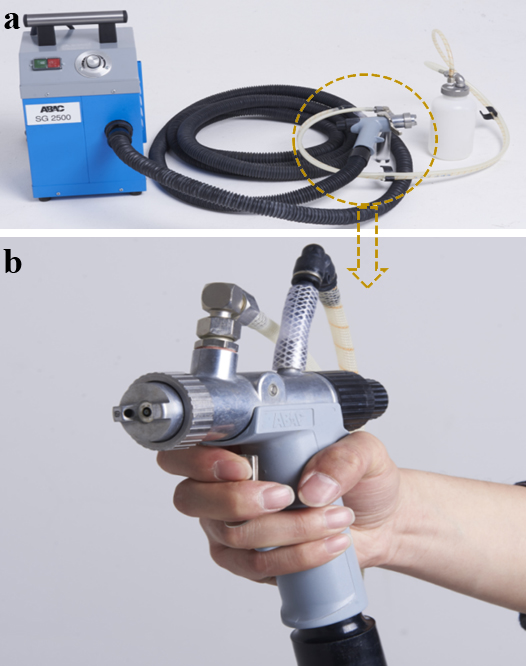


**Figure S16: Setup for facile preparation water soluble graphene coating.**





**Figure S17: SEM image of water soluble graphene film on PET substrate.**

**
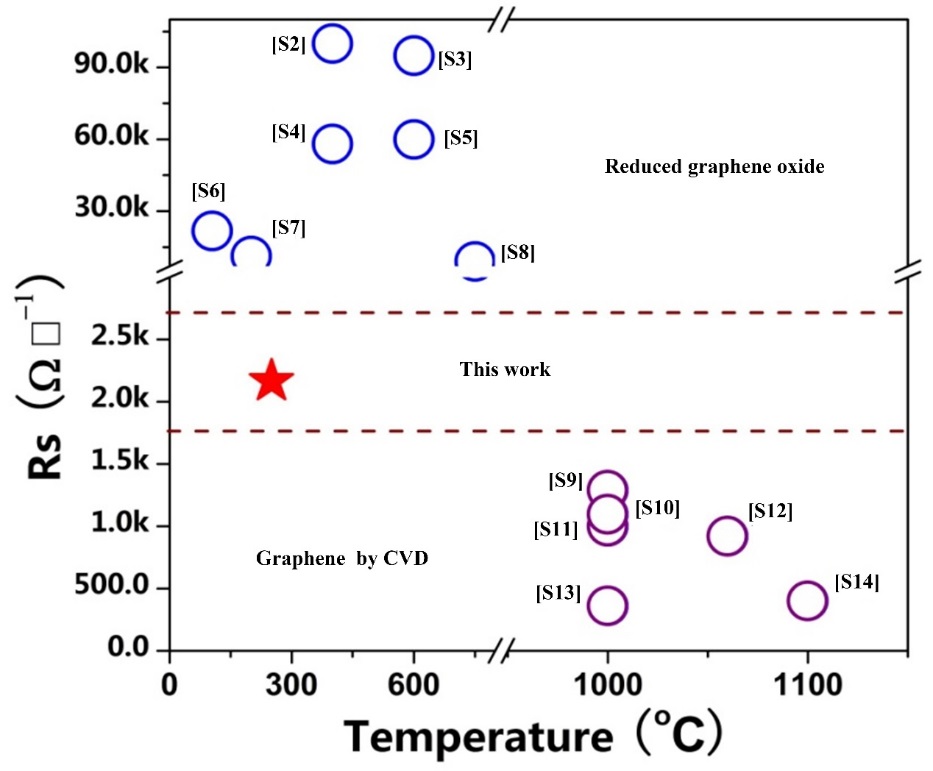
**

**Figure S18: Sheet resistances (****Rs) of water soluble graphene, compared with previously reported CVD graphene films and reduced graphene oxides.** The statistical information shows the Rs of most previously reported rGO ranges from 100000-20000 Ω □^-1^. The Rs of CVD graphene ranges from 50-1500 Ω/□. The Rs of water soluble graphene is 2100 Ω/□, which is close to that CVD graphene. Furthermore, the treating temperature is very low of the water soluble graphene.

**
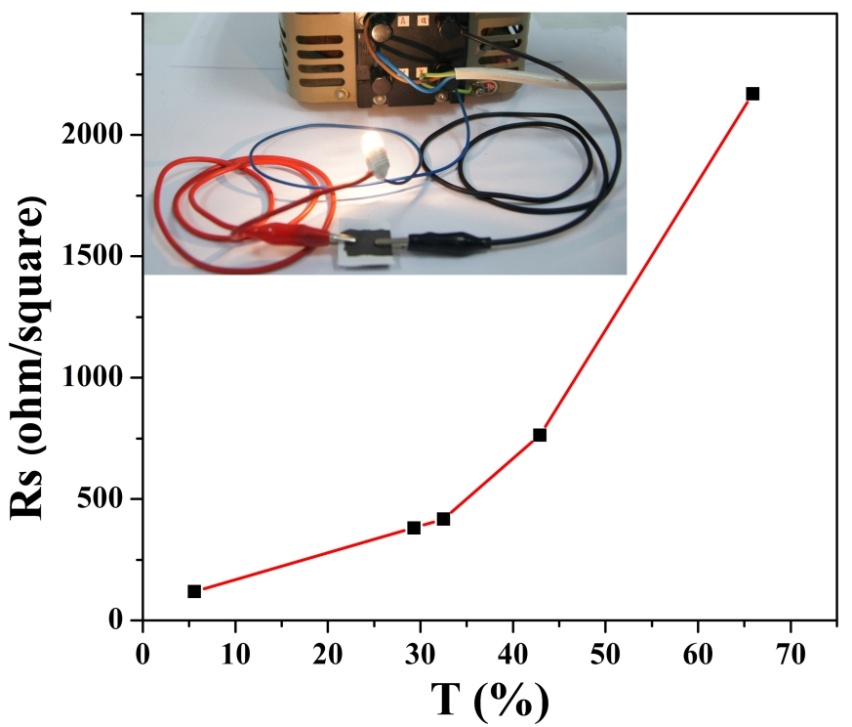
**

**Figure S19: The square resistance and transparency (450 nm visible light) of different amount of water soluble graphene coating on PET.**


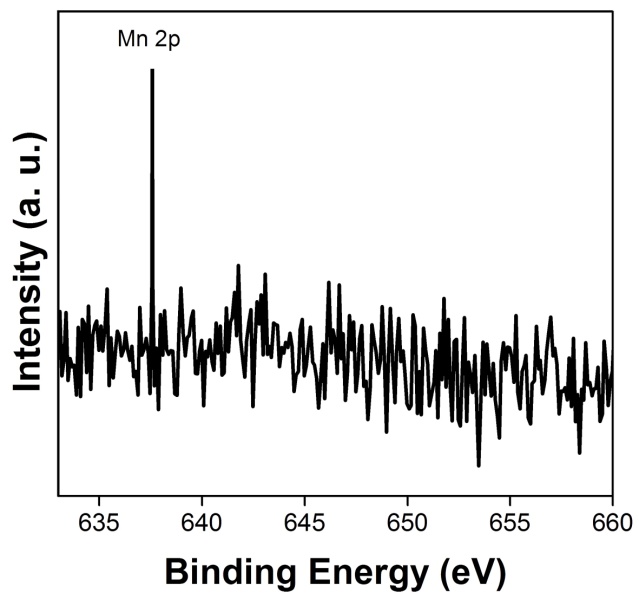


**Figure S20: Mn 2p XPS spectra of precursor. The peak located at 638.2 eV can be due to the Mn(III).**


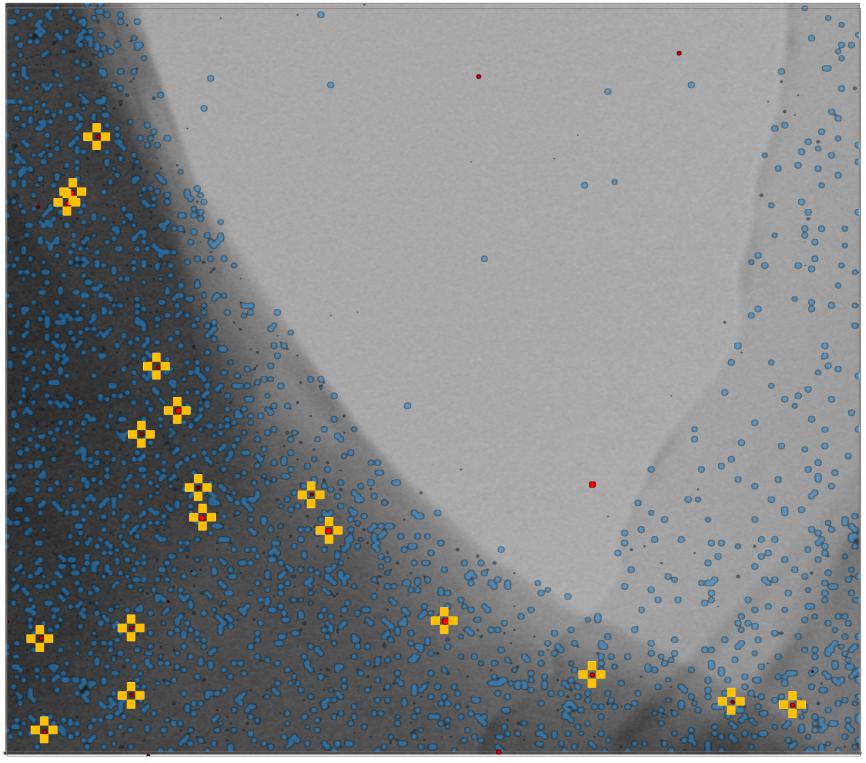


**Figure S21: EDS mapping image under TEM of edge oxidized graphite.** The blue and yellow point corresponds to C and Mn, respectively.

**
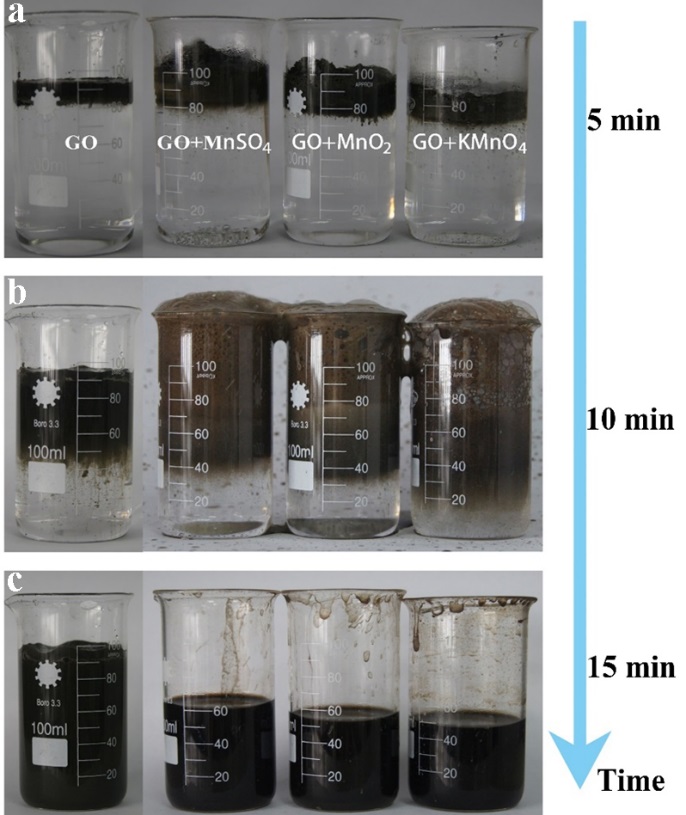
**

**Figure S22:** Exfoliation of GO, GO with MnSO4, GO with MnO2, and GO with KMnO4 in 80 mL mixture of NH_3_: H_2_O_2_: H_2_O with volume ratio of 1:4:5 under different time. (a) 5 min, (b) 10 min, and (c) 15 min.


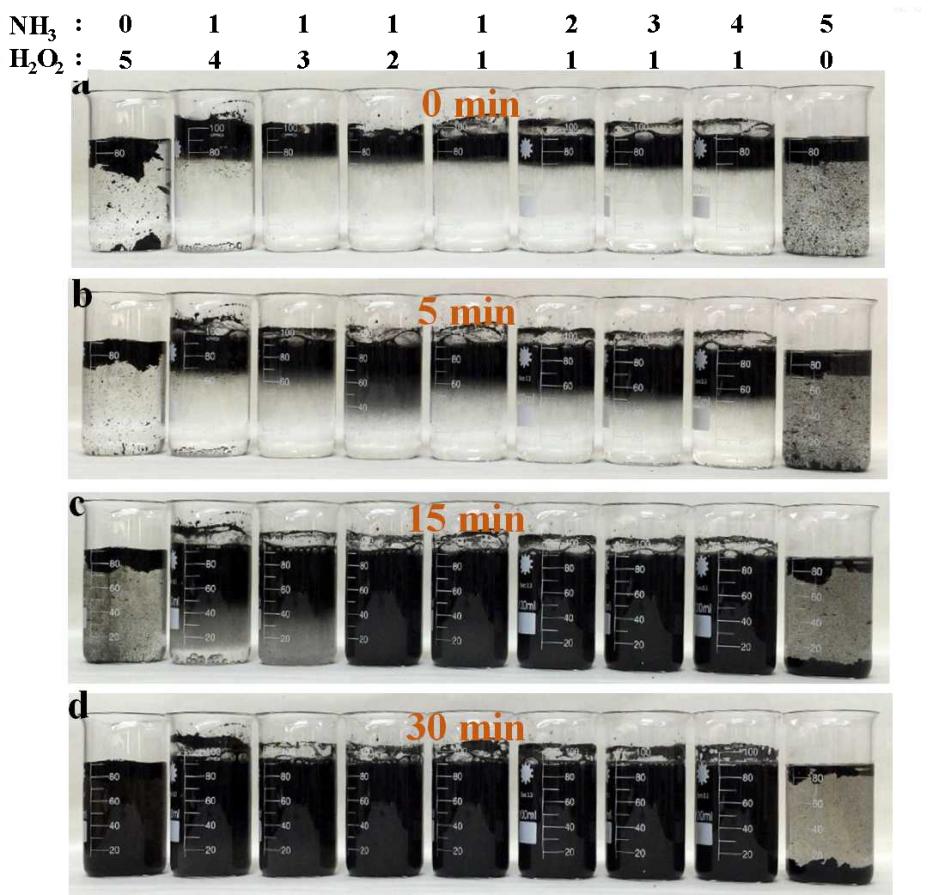


**Figure S23:** Exfoliation progress of edge oxidized graphite in different mixed solutions (volume of NH_3_: 0, 1, 1, 1, 1, 2, 3, 4 and 5 units from left to right; volume of H_2_O_2_: 5, 4, 3, 2, 1, 1, 1, 1 and 0 units from left to right. 1 unit volume is 10 mL) under different time. (a) 0 min, (b) 5 min, (c) 15 min and (d) 30 min. The Video S2 shows the corresponding dynamic process.

**
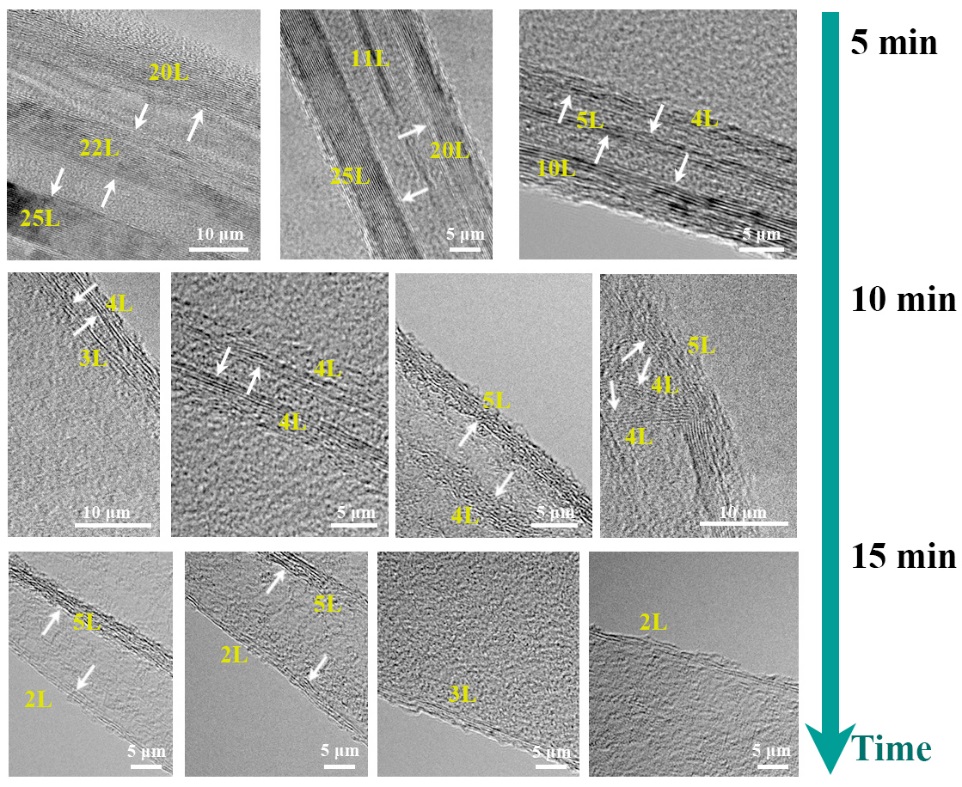
**

**Figure S24: The TEM images of the products after bubbling exfoliation with 5, 10 and 15 minutes, indicating the gradual dissociation of edge oxidized graphite.**


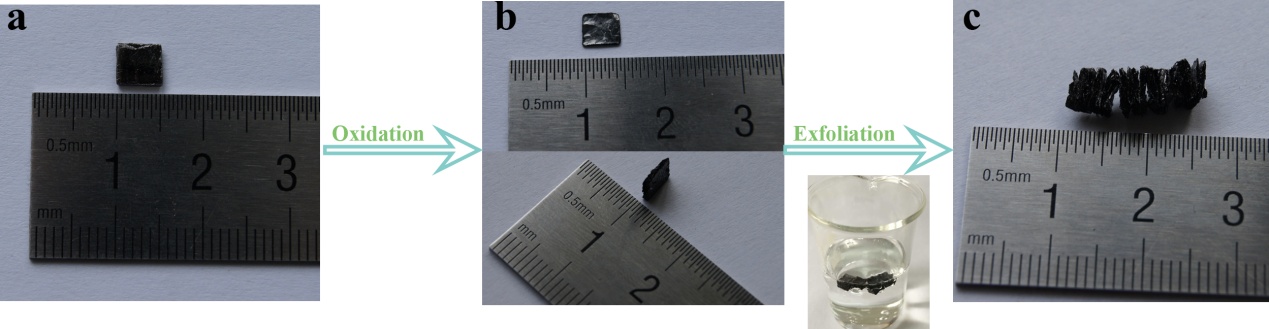


**Figure S25: Exfoliation progress of HOPG.** (a) digital photograph of a piece of HOPG. The length/width is 5.0 mm and the thickness is 0.8 mm. (**b**) digital photograph of edge oxidized HOPG, showing no obvious change with the raw material, which indicates there is no obvious exfoliation during the oxidation. (**c**) digital photograph of HOPG after bubbling exfoliation in the mixed solution of H_2_O_2_ (3.9 M) and NH_3_ (1.3 M). The interlayer catalytic exfoliation of the edge oxidized HOPG produces an expanded string consisting of the interconnected graphene layers.

**Video Legends**

Video 1: The typical bubbling process in the mixture of H_2_O_2_ and NH_3_

Video 2: The bubbling exfoliation with different H_2_O_2_ and NH_3­_ additions. From left to right, the volume of H_2_O_2_ is 5, 4, 3, 2, 1, 1, 1, 1 and 0 units, and the volume of NH_3_ is 0, 1, 1, 1, 1, 2, 3, 4 and 5 units, respectively (1 unit volume is 10mL).

Video 3: The bubbling exfoliation on HOPG sample in the mixture of 40 mL H_2_O_2_ and 10 mL NH_3_.

**Supplementary references**

1. Liu, G. *et al.* Low-frequency electronic noise in the double-gate single-layer graphene transistors. *Appl. Phys. Lett.* **95,** 033103–033103–3 (2009).
2. Becerril, H. A. *et al.* Evaluation of solution-processed reduced graphene oxide films as transparent conductors. *ACS nano* **3,** 463–470 (2008).
3. Yang, J. *et al.* Synthesis, characterization and optical property of graphene oxide films. *Applied Surface Science* **258,** 5056–5060 (2012).
4. Shi, H. F. *et al.* Transparent conductive reduced graphene oxide thin films produced by spray coating. *Sci China-Phys Mech Astron* **58,** 0142021–1–014202–5 (2015).
5. Sun, H. B., Yang, J., Zhou, Y. Z., Zhao, N. & Li, D. Preparation of reduced graphene oxide films by dip coating technique and their electrical conductivity. *Materials Technology: Advanced Performance Materials* **29,** 14–20 (2014).
6. Zhao, C. L. *et al.* Formation of uniform reduced graphene oxide films on modified PET substrates using drop-casting method. *Particuology* **17,** 66–73 (2014).
7. Zhu, Y. W., Cai, W. W., Piner, R. D., Velamakanni, A. & Rouff, R. S. Transparent self-assembled films of reduced graphene oxide platelets. *Applied Physics Letters* **95,** 103104 (2009).
8. Cheng, M. *et al.* Restoration of graphene from graphene oxide by defect repair. *Carbon* **50,** 2581–2587 (2012).
9. Yamada, T., Kim, J., Ishihara, M. & Hasegawa, M. Low-temperature graphene synthesis using microwave plasma CVD. *J. Phys. D: Appl. Phys.* **46,** 063001 (2013).
10. Liu, Y. L. *et al.* Study on temperature-dependent carrier transport for bilayer graphene. *Physica E* **69,** 115-120 (2015).
11. Kim, M. S., Woo, J. M., Geum, D. M., Rani, J. R. & Jang, J. H. Effect of copper surface pre-treatment on the properties of CVD grown graphene. *AIP Advances* **4,** 127107 (2014).
12. Strudwick, A. J. *et al.* Chemical vapor deposition of high quality graphene films from carbon dioxide atmospheres. *ACS nano* **9,** 31–42 (2015).
13. Pu, J. *et al.* Chemical vapor deposition growth of few-layer graphene for transparent conductive films. *RSC Adv.* **5,** 44142–44148 (2015).
14. Veronese, G. P. *et al.* Graphene as transparent conducting layer for high temperature thin film device applications. S*olar Energy Materials & Solar Cells* **138,** 35–40 (2015).
15. Kovtyukhova, N. I. *et al.* Layer-by-layer assembly of ultrathin composite films from micron-sized graphite oxide sheets and polycations. *Chem. Mater.* **11,** 771–778 (1999).
16. Hirata, M., Gotou, T., Horiuchi, S., Fujiwara, M. & Ohba, M. Thin-film particles of graphite oxide 1. *Carbon* **42,** 2929–2937 (2004).
17. Marcano, D. C. *et al.* Improved synthesis of graphene oxide. *ACS Nano* **4,** 4806–4814 (2010).
